# Characterization and comparative genomic analysis of novel lytic bacteriophages targeting *Cronobacter sakazakii*

**DOI:** 10.1016/j.virusres.2023.199102

**Published:** 2023-04-01

**Authors:** Yuan-Song Zhang, Lei Yuan, Fedrick C. Mgomi, Cao-Wei Chen, Yang Wang, Zhen-Quan Yang, Xin-an Jiao

**Affiliations:** a School of Food Science and Technology, Yangzhou University, 196 Huayang West Road, Yangzhou, Jiangsu 225127, PR China; b Jiangsu Key Laboratory of Zoonoses, Yangzhou, Jiangsu 225009, PR China

**Keywords:** *Cronobacter sakazakii*, Phage, Biocontrol agent, Genomics, Dairy products

## Abstract

*Cronobacter sakazakii,* a foodborne pathogen, can contaminate powdered infant formula (PIF) and cause life-threatening meningitis, necrotizing colitis and meningoencephalitis in infants. Bacteriophages are increasingly considered an efficient approach to target pathogenic microorganisms. In the current study, four virulent phages that can infect *C. sakazakii* were isolated from sewage samples, and their biological and complete genomic characteristics were analyzed. A comparative genomic analysis was performed to investigate the functional genes and phylogenetic evolution of the four phages. The results revealed that all four phages belonged to the *Ackermannviridae* family. Notably, the viral burst size of the phages ranged from 10 to 250 PFU/cell, following a latent period of 5 min to 20 min. Moreover, phages were stable over a pH range of 4 to 10 and a temperature range of 50 ℃ to 60 ℃. The full length of the complete genomes of the four phages ranged from 41,929 bp to 146,806 bp, containing lysis genes but no virulence genes. Phylogenetic tree analysis showed that the four phages were members of two distinct genetic groups with a significant genetic evolutionary distance between each *C. sakazakii* phage. Furthermore, the antibacterial assay revealed that all phages could inhibit the growth of *C. sakazakii* for up to 24 h. Taken together, the four phages have huge prospects as additives in dairy products to counter *C. sakazakii*.

## Highlights

• Four new lytic phages infecting *Cronobacter sakazakii* were isolated from sewage samples.
• Morphological, physiological, genomic characteristics of phages were characterized.
• *C. sakazakii* phage origin, evolution and their connections were studied by a comparative genomics analysis.

## 1 Introduction

*Cronobacter sakazakii* is an emerging and opportunistic foodborne pathogen that can lead to bacteremia, meningitis, and necrotizing enterocolitis, with case fatality rates ranging from 50% to 80% (Ji et al., 2021; Ling et al., 2020, 2021). This pathogen has been detected in various food products, including powdered infant formula (PIF), and represents a concern in food processing environments (Pei et al., 2019). Given the antimicrobial resistance of *C. sakazakii*, targeting this pathogen could be challenging (Henry and Fouladkhah, 2019), emphasizing the importance of developing safe and effective biocontrol agents to manage *C. sakazakii* infection.

Bacteriophages (phages) are natural viruses that infect bacterial cells. They replicate, induce bacterial cell lysis, and eventually release their progeny. Phages are widespread in the biosphere and harmless to animals and humans (D'Accolti et al., 2021). Owing to their high specificity, phages only lyse a certain host bacteria. Although several studies on *C. sakazakii*-infecting phages, including PF-CE2 (Xiao et al., 2021), PBES04 and PBES19 (Kim et al., 2021), JK004 (Wang et al., 2022) and EspYZU01 - EspYZU10 (Li et al., 2021b), have been reported in recent years, they only focused on single aspects, such as whole genome sequencing (WGS), general physiological properties, and their roles as biocontrol agents during the processing of milk, cheese and other dairy products. However, there is still a lack of comprehensive research on *C. sakazakii* phages, including their physiological properties and bioinformatics analysis. More comparative genomics analyses are required to explore the diversity and complex relationships among different *C. sakazakii* phages to understand their biological characteristics, genetic diversity and evolution, providing new guidance for the effective use of phages for pathogen control.

Herein, four lytic phages were uncovered in sewage samples collected from Yangzhou, China, and further phage morphology and biological analyses were conducted. Furthermore, their genomic characteristics and genealogy were investigated to offer novel insights into *C. sakazakii* lysogenic phage populations and assess their diversity.

## 2 Materials and methods

### 2.1 Bacterial strains and culture conditions

The ten *C. sakazakii* strains used in this study (Table S1) were incubated overnight in a nutrient broth (NB, Haibo Biotechnology Co., Ltd. Qingdao, China) and a 120 rpm shaking incubator. *Escherichia coli* 10,664, *Salmonella* Enteritidis 21,513, *Staphylococcus aureus* 21,600, and *Bacillus cereus* 21,261 (Table S1) were cultured in a Luria-Bertani medium (LB) (Huankai Microbial Sci & Tec. Co. Ltd. Guangzhou, China) in a shaking incubator at 37 °C and 120 rpm overnight.

### 2.2 Isolation and purification of *C. sakazakii* phages

Forty sewage samples (100 mL of each) were collected from various sewages in Yangzhou, China (Table 1). The isolation and purification method of *C. sakazakii* phages was performed as previously described by Chen et al. (2022), with minor modifications. To remove solid particles, ten milliliters of each sample were centrifuged at 8000 *g* for 10 min and filtered through 0.45-μm-pore-size syringe filters (Millex, Merck Millipore Ltd., Ireland). Thereafter, 100 μL of each *C. sakazakii* strain was combined with 5 mL of each filtrate in 5 mL 2 × NB for incubation at 37 °C for 12 h with continuous shaking at 120 rpm. The incubated cultures were subsequently centrifuged at 8000 *g* for 10 min, and the supernatants were filtered through a 0.22-μm-pore-size syringe filter (Millex, Merck Millipore Ltd., Tullagreen, Carrigtwohill, Co. Cork, Ireland). The filtrates were collected and preserved at 4 °C for the ensuing experiments.

**Table 1:** Characterization of *Cronobacter* phages by plaque morphology and transmission electron microscopy.

Phage	Host strain	Source	Plaque diameter (mm)	Morphological analysis by TEM^a^		Presumptive family
				Head (diameter)	Tail (length)	
EspYZU12	*C. sakazakii* CICC 22,919	Domestic sewage	0.33	Isometric (82 nm)	106 nm, with contractile tail sheath	*Ackermannviridae*
EspYZU13	*C. sakazakii* CICC 21,569	Farmers’ market sewage	4.81	Isometric (74 nm)	102 nm, with contractile tail sheath	*Ackermannviridae*
EspYZU14	*C. sakazakii* CICC 22,919	Kitchen sewage	0.26	Isometric (82 nm)	105 nm, with contractile tail sheath	*Ackermannviridae*
EspYZU15	*C. sakazakii* CICC 22,919	Cattle farm sewage	0.19	Isometric (67 nm)	121 nm, with contractile tail sheath	*Ackermannviridae*

a Transmission electron microscopy.

The filtrates were utilized to test for the presence of novel lytic phages by plaque development on double-overlay agar (Chen et al., 2021). Briefly, 100 μL of the filtrate was mixed with 100 μL of each overnight culture of *C. sakazakii* in 5 mL of nutrient soft agar (0.7% agar), poured onto nutrient agar plates (1.5% agar), and then incubated for 10 h at 37 °C until clear plaques were observed. A single and clear plaque was selected, re-amplified, and selected again. The process was repeated at least three consecutive times for purified phages. Next, a total of 100 μL of each purified phage was diluted with SM buffer (8 mM MgSO_4_∙7H_2_O, 100 mM NaCl, 5 mL/L of 2% gelatin solution, and 50 mM Tris–HCl, pH 7.5) by a series of ten-fold dilutions, and mixed with 100 μL *C. sakazakii* (10^7^ CFU/mL), to form nutrient agar plates as described above. The phage titer was expressed as plaque-forming units per milliliter (PFU/mL).

### 2.3 Morphological characteristics of phages

The phage morphology was visualized under a transmission electron microscope (TEM, Tecnai 12, Tecnai, Eindhoven, the Netherlands). High phage titers were concentrated using cesium chloride (CsCl, Sangon Biotech Co., Ltd, Shanghai, China) density gradient centrifugation and 10% polyethylene glycol (PEG) 8000 (Sangon Biotech Co., Ltd, Shanghai, China) precipitation, as previously described (Byun et al., 2022). Afterward, 30 μL of concentrated phage suspension was applied to a 400-mesh copper grid with a membrane and allowed to adsorb for 10 min; a filter paper was used to absorb excess liquid around the copper mesh. Then, the resulting product was dyed with 2% phosphotungstic acid (PTA, Aladdin Biochemical Technology Co., Ltd, Shanghai, China) for 10 min, and excess PTA was blotted off the filter paper and dried out under an infrared lantern.

### 2.4 Characterization of isolated phages

#### 2.4.1 Determination of phage lysis spectrum

The phage lytic spectrum was assessed using the spot-on-lawn antimicrobial assay (Nafarrate et al., 2021). Ten microliters of each strain (10^7^ CFU/mL) were dropped onto the solid plate and left for 15 min. Next, 2 μL of purified phage (10^8^ PFU/mL) was overlaid on the bacterial lawn and incubated for 8 h at 37 ℃. Phage plaque formation indicated the presence of lytic activities.

#### 2.4.2 Thermal and pH stability test

This procedure was conducted to test phage stability under various temperatures and pH conditions, as described by Li et al. (2022). A total of 2 mL of phages (10^8^ PFU/mL) were incubated at different temperatures (50 ℃, 60 ℃, 70 ℃, and 80 ℃) with continuous shaking at 200 rpm for 60 min. Then, each 500 μL of phages was taken out at 20 min, 40 min, and 60 min. To evaluate the stability of the phages under different pH values, 100 μL of purified phage particles (10^8^ PFU/mL) were incubated in 900 μL of NB, adjusted to a pH value ranging from 2 to 12, at 37 °C for 2 h with continuous shaking. Following incubation, the samples were analyzed for titer (PFU/mL) using the double-layer agar plate method described in Section 2.2. Phage survival rate = (phage titer after incubation at different pH values) / (phage titer after incubation at pH 7) × 100%.

#### 2.4.3 Optimal multiplicity of infection

The multiplicity of infection (MOI) refers to the ratio of added bacteriophages to the number of host bacteria at the time of infection (Li et al., 2021a). 100 μL of overnight host bacterial *C. sakazakii* (10^5^ CFU/mL) cultures were infected with 100 μL of phages at different ratios (0.001, 0.01, 0.1, 1, and 10 PFU/CFU) in 5 mL of NB and incubated at 37 °C for 12 h with continuous shaking. To eliminate bacterial debris, the phage was filtered through a 0.22-μm-filter membrane after centrifugation at 8000 *g* at 4 °C for 10 min. The optimal MOI corresponded to the highest titer.

#### 2.4.4 One-step growth curve experiments

A total of 100 μL of purified phage was mixed with overnight-cultured host strain at the optimal MOI in 5 mL of NB. The culture was centrifuged at 4 °C (8000 *g* for 10 min) after incubation at 37 °C for 10 min. The residue was then washed twice to remove non-infected phages, following which it was placed in 5 mL of fresh NB and incubated for 120 min at 37 °C. Phage titer was determined after collecting 100 μL samples every 10 min for 60 min and then every 20 min for the next 120 min. The one-step growth curve was plotted using the infection time (min) as the abscissa and phage titer as the ordinate (PFU/infected cells). The growth properties of phages, such as latent time, burst period, and burst size, were determined using one-step growth curves (Wan et al., 2021).

### 2.5 The effect of phages as biocontrol agents against *C. sakazakii*

The effect of phages as biocontrol agents against *C. sakazakii* was investigated as previously described by Luo et al. (2021) with minor modifications. Briefly, the NB was artificially infected with *C. sakazakii* at a concentration of 10^4^ CFU/mL at room temperature for 10 min for attachment. Then, the samples were mixed with phages at MOI of 100, 10, 1, 0.1, and 0.01, respectively, and phage-free samples served as the control. All samples were incubated at 37 ℃ for 24 h with continuous shaking. The mixtures of *C. sakazakii* were cultured in an automated microplate reader (Infinite® F50, TECAN, Switzerland), and the bacterial cell density (OD_600_) of each mixture was determined at 1-h intervals. The maximum phage inhibition value (I_max_) was calculated as follows: (OD_600_ value of the phage-free group) - (OD_600_ value of the different MOI groups).

### 2.6 Genomic analysis of phages

The extraction and purification of the genomic DNA of phages were conducted according to the instructions of the Lambda Phage Genomic DNA Kit (ZP317, Zoman Biotechnology Co., Ltd., Beijing, China). Total phage DNA integrity was assessed using 2% agarose gel electrophoresis and quantified with TBS 380 (Invitrogen, Carlsbad, CA, USA). The Illumina NovaSeq 6000 platform was employed for whole-genome sequencing (Illumina, San Diego, CA, USA). The original sequences were trimmed and optimized by the Trimmomatic-0.39 and ABySS software, respectively, and genome assembly and coding gene prediction were performed using the GapCloser (version 1.12) and GeneMarkS software (version 4.28). In addition, the protein functions of the newly determined genes were compared with Basic Local Alignment Search Tool (BLAST) NR, Swiss-Prot, eggNOG, KEGG, and GO databases (comparison criteria: E value ≤ 1e-5). The National Center for Biotechnology Information Search database (NCBI, https://www.ncbi.nlm.nih.gov/) was used for sequence alignment, and MAGA X software and iTOL software (version 2.1) were used to construct and embellish the phylogenetic tree.

### 2.7 Statistical analysis

All tests were independently performed in triplicates, and the data were presented as mean ± standard deviation. Data analysis was performed using SPSS 24.0 software (SPSS Inc., Chicago, IL, USA), and *p* < 0.05 was considered statistically significant.

## 3 Results and discussion

### 3.1 Isolation and morphological characteristics of phages targeting *C. sakazakii*

**Fig. 1:**
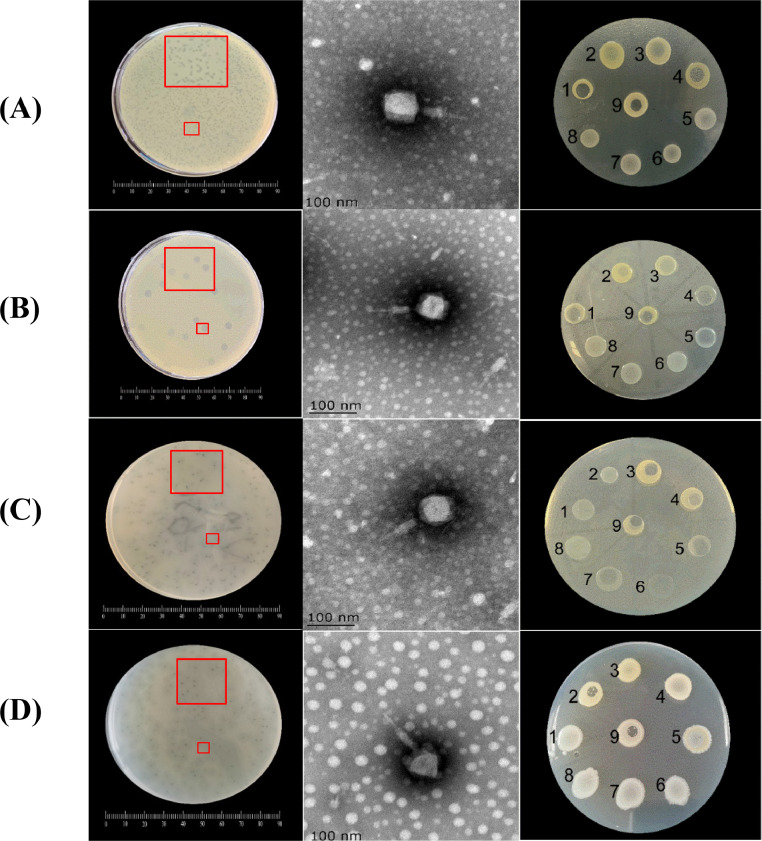
Plaques of the phage on the host plate. Electron micrographs of phages and the lysis spectrum of phages: No.1, *C. sakazakii* 21,560, No.2, *C. sakazakii* 21,545, No.3, *C. sakazakii* 21,673, No.4, *C. sakazakii* 21,569, No.5, *E. coli* 10,664, No.6, *S*. Enteritidis 21,513, No.7, *S. aureus* 21,600, No.8, *B. cereus* 21,261, No.9, *C. sakazakii* 22,919. (A) EspYZU12. (B) EspYZU13. (C) EspYZU14. (D) EspYZU15. Each bar represents 100 nm.

Phages are ubiquitous and abundant in natural environments, with an estimated 4.8 × 10^31^ phage particles (Moura de Sousa et al., 2021). In this study, four phages (EspYZU12, EspYZU13, EspYZU14, and EspYZU15) with the ability to infect *C. sakazakii* CICC 22,919 and CICC 21,569 were isolated from sewage samples in Yangzhou, China (Table 1). These four phages formed clear plaques on their host plates, varying from 0.19 mm to 4.81 mm in diameter (Fig. 1).

Phage morphology was visualized using TEM (Fig. 1). It was found that all four phages had an isometric head (diameter ranging from 67 nm to 82 nm) and tail (diameter ranging from 102 nm to 121 nm), and a contractile tail sheath. Based on these morphological characteristics, the phages were attributed to the *Caudoviricetes* order. According to the classification rules recommended by the Ninth Report of the International Committee on Taxonomy of Virus (ICTV), the four phages belong to the *Ackermannviridae* families. In a previous study (Li et al., 2021b), *C. sakazakii* phages were classified into the *Demerecviridae* and *Autographiviridae* families, indicating the potential morphological diversity of *C. sakazakii*-infecting phages*.*

### 3.2 Lysis spectrum of phages

The study of a selection of broad-spectrum lytic phages could considerably clarify the use and application of phages as biocontrol agents. The plaque assay was employed to investigate the effect of the four phages on 14 bacterial strains, including *E. coli* 10,664, *S.* Enteritidis 21,513, *S. aureus* 21,600, *B. cereus* 21,261, and 10 *C. sakazakii* strains (Table S2). EspYZU14 exhibited lytic activity against 5 isolates of *C. sakazakii* with relatively high specificity (35.71%, 5/14), while the remaining three phages could lyse more than 6 strains. EspYZU13 exhibited the broadest lytic spectrum with 9 out of 14 strains (64.29%). However, as expected, the phages could not lyse *E. coli* 10,664, *S.* Enteritidis 21,513, *S. aureus* 21,600, and *B. cereus* 21,261 (Fig. 1). The strict host range properties of these phages are considered key points emphasizing their potential as good targeting candidates in food applications (Cong et al., 2021). The results demonstrated that the lytic abilities of the phages against the strains were not influenced by their origin, which is inconsistent with findings of another study (Yang et al., 2019).

### 3.3 pH and thermal stability of phages

**Fig. 2:**
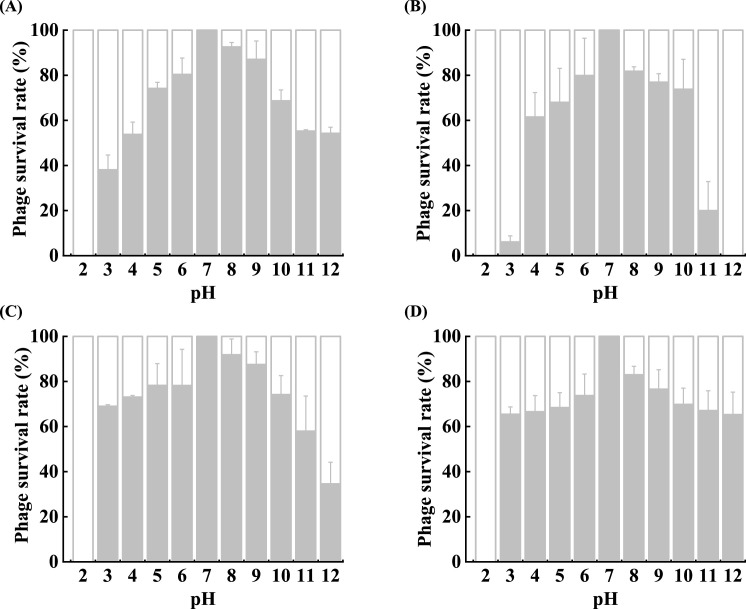
Resistance of the phages to acidity and alkalinity. The four phages were incubated under different pH conditions for 2 h. (A) EspYZU12. (B) EspYZU13. (C) EspYZU14. (D) EspYZU15.

**Fig. 3:**
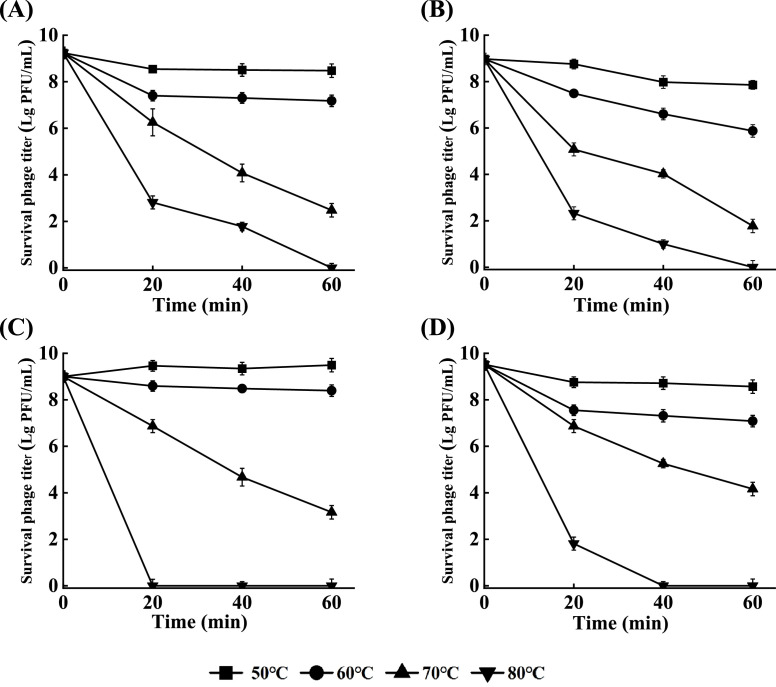
Thermal stability tests of the four phages. The four phages were incubated at different temperatures (50 ℃, 60 ℃, 70 ℃, and 80 ℃), and survival phage titers were measured at 20-min intervals for 60 min. (A) EspYZU12. (B) EspYZU13. (C) EspYZU14. (D) EspYZU15.

Measuring the tolerance of phages to environmental conditions is a critical step for assessing their potential applications in the industry. In this study, the phage survival rate decreased by varying amounts at a pH above or below 7. We found that EspYZU12, EspYZU14, and EspYZU15 were stable at pH values ranging from 5 to 10 after 2 h incubation at 37 ℃. In contrast, the phage EspYZU13 could maintain high activity within a narrow pH range of 4 to 10 (Fig. 2). The thermal stability of phages was assessed by exposing them to temperatures of 50 °C, 60 °C, 70 °C, and 80 °C for 60 min, with the inactivation curves illustrated in Fig. 3. All four phages remained highly active (> 10^6^ PFU/mL) after 60 min of incubation at 50 ℃ and 60 ℃. After treatment at 70 ℃ for 60 min, the phages remained highly active (10^2^ - 10^4^ PFU/mL). When the temperature reached 80 ℃, only EspYZU12 and EspYZU13 maintained their activities after 40 min of treatment, whereas EspYZU14 and EspYZU15 were completely inactivated. Consistently, the thermal stabilities of EspYZU12 and EspYZU13 have been reported to be higher than other *C. sakazakii* phages in the literature.

It is well documented that the physicochemical factors pH and temperature may impact the survival and stability of phages. Other phages, such as JK004, have been documented to exhibit significant stability over a pH range of 6 to 8 and temperature of 30 ℃ to 40 ℃. However, the phages in the current study showed a wide pH (pH 5 - 10) and thermal stability range (50 ℃ to 70 ℃) (Endersen et al., 2017; Wang et al., 2022). These properties benefit phages by playing a role as a biorecognition element, which could be combined with chemical analysis approaches to design a chemical biosensing method to capture viable *C. sakazakii* cells (Nakama et al., 2021).

### 3.4 The optimal multiplicity of infection (MOI)

**Fig. 4:**
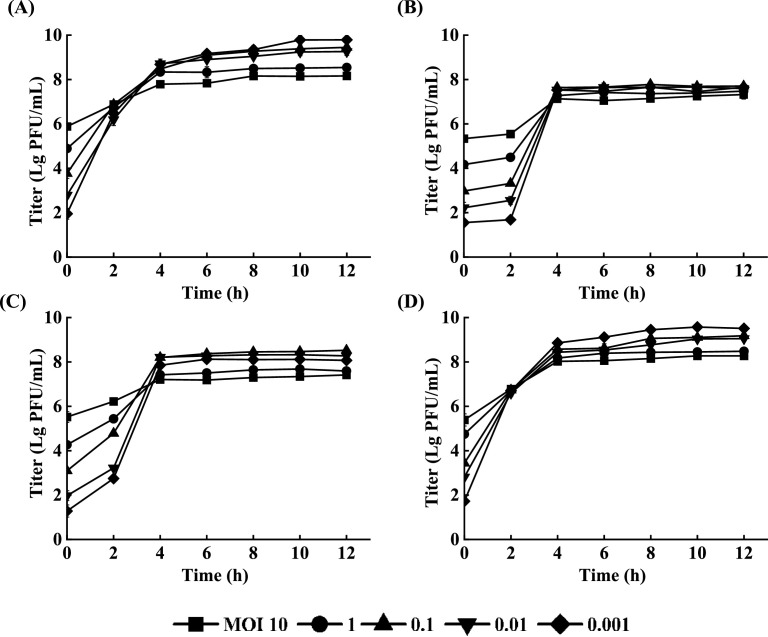
The determination of the multiplicity of infection of the four phages. The culture of *C. sakazakii* (10^5^ CFU/mL) was infected with four phages under different MOI (10, 1, 0.1, 0.01, and 0.001) at 2-h intervals for 12 h. (A) EspYZU12. (B) EspYZU13. (C) EspYZU14. (D) EspYZU15.

The dynamics of the phage titer curves with different MOIs are depicted in Fig. 4. All four phages displayed a marginal increase in potency within 2 h, with an accelerated increase after 2 h. The peak titers of phages EspYZU13 and EspYZU14 were observed (10^7^ - 10^8^ PFU/mL) at 4 h, and the ideal MOI for phage EspYZU13 and EspYZU14 was 0.1. In addition, the peak titers of phages EspYZU12 and EspYZU15 (10^9^ PFU/mL) were observed at 10 h maintained over the next incubation period, with an optimal MOI of 0.001. It has been established that the optimal MOI for the isolated *C. sakazakii* phage was 0.01 (Wang et al., 2022). The lower initial inoculation amount of the four phages (not less than 10^4^ PFU/mL) enabled sufficient contact between the phage and the host to ensure its survival. Finally, the phage could achieve effective proliferation and generate large numbers of progeny bacteriophages (Wang et al., 2019).

### 3.5 One-step growth curve of phages

**Fig. 5:**
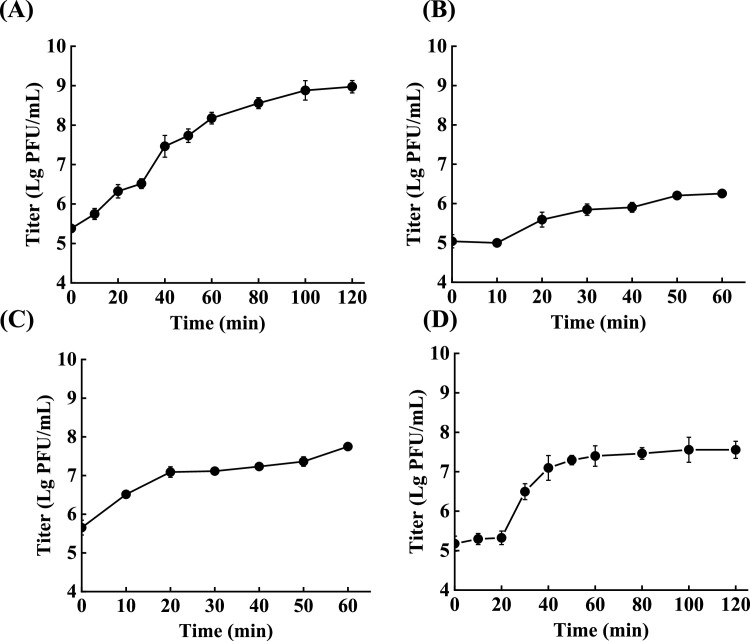
One-step growth curve of the four phages. Phage suspensions were collected at 10-min intervals for up to 60 min, followed by 20-min intervals for up to 120 min. (A) EspYZU12. (B) EspYZU13. (C) EspYZU14. (D) EspYZU15.

Fig. 5 depicts the one-step growth curve of the four phages in *C. sakazakii* hosts. The findings revealed that the latent period of phages EspYZU12 (EspYZU14) and EspYZU13 were 5 min and 10 min with a burst size of 10 to 250 PFU/host cell. Additionally, the infection of *C. sakazakii* by EspYZU15 displayed a latent period of 20 min and a burst period of 60 min with a burst size of 96 PFU/host cell. The latent period and burst size are vital factors that determine the selection of a phage as a biocontrol agent. It is widely thought that phages with short latency and large burst sizes can efficiently inactivate bacteria (Yazdi et al., 2020). Compared to other reported *C. sakazakii* phages, such as phages EspYZU01 and CS01 (Kim et al., 2019; Li et al., 2021c), which showed burst sizes of 65 PFU/host cell and 90.7 PFU/host cell and latent periods of 20 min and 60 min, respectively, three of the four phages presented a shorter latent period and larger burst size, which may increase the *C. sakazakii* lysis efficiency of the four phages.

### 3.6 Application of phages as biocontrol agents against *C. sakazakii*

**Fig. 6:**
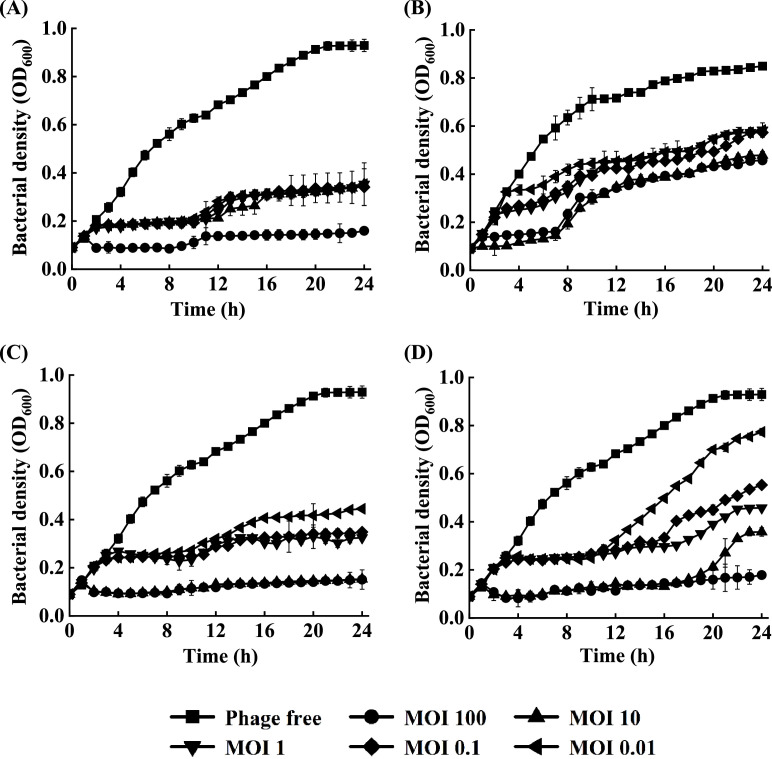
Bacteriostatic effect of the four phages. The phages were treated against *C. sakazakii* (10^4^ CFU/mL) at different MOI (100, 10, 1, 0.1, and 0.01) for 24 h. Phage-free represents positive control (no phage treatment). Bacterial density (OD_600_) was recorded at 1-h intervals for 24 h. (A) EspYZU12. (B) EspYZU13. (C) EspYZU14. (D) EspYZU15.

To assess the potential of the phages as biocontrol agents, their respective physiological properties (Table S3) and inhibitory effects on *C. sakazakii* were examined. The four phages exerted an inhibitory effect against *C. sakazakii* at 37 ℃ for 24 h. The OD_600_ value of the phage-free group increased from 0.08 to 0.92, with the highest inhibitory capacity observed for EspYZU12 (Fig. 6) at an Imax value of 0.77 in the MOI 100 treatment group and different inhibitory energies (Imax = 0.58 - 0.77) in all five treatment groups with MOIs of 0.01 - 100. Furthermore, the inhibitory effect was MOI-dependent, which supports the hypothesis that when a sufficient number of phage particles adhere to the cell, they lyse viable cells mediated by the activity of endolysin enzymes, even though they are sparse host groups (Kumar et al., 2020). In addition, endonuclease restriction in host bacteria may confer natural resistance in phage-host interactions (Kitti et al., 2022). For instance, the bacterial density of *C. sakazakii* was inhibited by phage EspYZU13 treatment for 6 h with an Imax value of 0.19 to 0.42 (Fig. 6B). Nonetheless, the growth of *C. sakazakii* in all treatment groups increased, which may be attributed to the fact that bacteria developed resistance to phages. For example, the bacterial host could prevent the phage from penetrating the host cell for DNA injection and replication by altering the phage binding site on the cell surface (Chevallereau et al., 2022) or cutting off the homologous sequence carried by the phage when it reinfects the bacteria by inserting a phage-derived sequence (called a spacer) into the bacterial CRISPR-Cas locus (Alseth et al., 2019). Typically, a large number of phages are required to improve infection efficiency based on the fact that elimination is associated with the "lysis from without" phenomenon, whereby a large number of phages are adsorbed on the cell surface, causing the cell to directly rupture and die, potentially delaying or preventing the emergence of resistance in host bacteria (Anjay et al., 2022).

### 3.7 Genomic analysis of phages

#### 3.7.1 Genome composition

The complete genome sequences of all four *C. sakazakii* phages have been uploaded to the NCBI GenBank database under accession numbers OP819284 (EspYZU12), OP819285 (EspYZU13), OP850600 (EspYZU14) and OP866730 (EspYZU15), respectively. The Illumina NovaSeq 6000 platform and GapCloser (version 1.12) software were used to sequence and assemble the complete genomes of the four lytic phages. The essential genome metrics (genome size, G + C content, and the number of predicted ORFs) of the four phage genomes were provided in Supplementary Fig. S1 and Table S4. The genomes consisted of double-stranded DNA ranging from 41,929 bp to 146,806 bp, with a G + C content of 46.71% to 54.71%, and several genes ranging from 45 to 239 bp (average gene length from 551 bp to 868 bp), representing 89.8% to 93.2% of the complete genome (Table S4). Interestingly, it has been reported that phages infecting the same host (bacteria) exhibit differences in genome size, which may be attributed to the number of genes encoding the relevant functions (Fong et al., 2019). Herein, the whole genome sequencing results support the notion that the genome size of the EspYZU13 (18 functional genes) was considerably smaller than the other three phages (39 - 40 functional genes). Similar findings were observed in phages LSA2308 and LSA2366; the genome of LSA2308 encoding 203 functional genes was 127,536 bp larger than LSA2366, which contained only 21 ORFs (Ma et al., 2021). In addition, four phages exhibited minimal homology with the other *Cronobacter* phage genomes in GenBank of *Cronobacter* phage vB_CsaM_GAP31 (with 84.37% homology and 69% coverage with EspYZU12), *Cronobacter sakazakii* phage vB_CskP_GAP227 (84.37% homology and 85% coverage with EspYZU13), *Cronobacter* phage CR9 (76.26% homology and no coverage with EspYZU14), *Cronobacter* phage PBES 02 (79.06% homology and 1% coverage with EspYZU15), implying that these four phages are novel lytic *Cronobacter* phages.

#### 3.7.2 Gene function annotation

Of the ORFs that had high similarities to sequences in the GeneBank databases, putative functions were assigned to 25% to 46% of the ORFs in the four phages in this study. A total of 198 out of 238 predicted ORFs were assigned to hypothetical proteins with unknown functions, possibly attributed to the limitations of the database used for annotation and the homogeneous number of phages (Fig. S2).

The annotated ORFs from the four phages were categorized into five functional groups: structure, lysis, DNA packaging, DNA manipulation, and others. Ten structure-related genes, including the conserved tail fiber protein, were common to all four phages (Table 2). Nevertheless, several proteins were present only in Group I or II phages. For example, the proteins structurally associated with Group I phages were identified as phage tail genes, which might benefit the adsorption and infection of the host bacterium. Group I phages might possess a layer of lipoprotein membrane on the surface because of unique membrane proteins. Major capsid protein, tail tubular protein A, and tail tubular protein B were exclusively present in Group II phages, coding for the phage head and tail, respectively. These three proteins constitute a complete phage particle mediating unique tail fiber assembly, head-tail connector, and scaffolding protein junction assembly. The adsorption of the tail fiber initiated the lytic mechanism of the Group II phages to the bacterial surface receptor (Pellizza et al., 2020). More importantly, the four phages in Groups I and II encode different tail fiber proteins. They exhibited distinct differences during lysis, allowing them to recognize other receptors in the different host strains, and yielded variable lysis effects on the *C. sakazakii* strains (Ahamed et al., 2019; Gudlavalleti et al., 2020).

**Table 2:** Functional groups of genes in the four phages.

Functional group	gene	Group I		Group II	
		EspYZU12	EspYZU14	EspYZU15	EspYZU13
Structure	major capsid protein	**–**	**–**	**–**	●
	tail fiber assembly protein	●	●	●	**–**
	conserved tail fiber protein	●	●	●	●
	tail sheath protein	●	●	●	**–**
	tail fiber protein	●	●	●	**–**
	head-tail connector protein	**–**	**–**	**–**	●
	scaffolding protein	**–**	**–**	**–**	●
	tail tubular protein A	**–**	**–**	**–**	●
	tail tubular protein B	**–**	**–**	**–**	●
	putative membrane protein	●	●	●	–
DNA Packaging	terminase large subunit	★	★	★	**–**
	putative baseplate assembly protein	●	●	●	**–**
DNA manipulation	DNA replicative helicase/primase	★	★	★	★
	DNA polymerase	★	★	★	★
	DNA ligase	★	★	★	**–**
	nicotinamide-nucleotide adenylyl transferase	★	★	★	**–**
	putative thymidylate synthase	★	★	★	**–**
	nicotinamide phosphoribosyl transferase	★	★	★	**–**
	tRNA nucleotidyl transferase	★	★	★	**–**
	putative phosphoribosyl pyrophosphate synthetase	★	★	★	**–**
	EndoVII packaging and recombination endonuclease	**–**	★	★	**–**
	DNA maturase B	**–**	**–**	**–**	★
	ATP-dependent DNA ligase	**–**	**–**	**–**	★
	tRNA nucleotidyltransferase/poly(A) polymerase	**–**	**–**	**–**	★
	DNA exonuclease	**–**	**–**	**–**	★
	DNA endonuclease VII	**–**	**–**	**–**	★
	DNA-dependent RNA polymerase	**–**	**–**	**–**	★
	putative integrase	★	★	★	**–**
Lysis	lytic glycosylase	**–**	**–**	**–**	★
	Holin	**–**	**–**	**–**	★
	Endolysin	**–**	**–**	**–**	★
	putative colanic acid degrading protein	★	★	★	**–**
Additional functions	putative carbohydrate binding domain protein	●	●	●	**–**
	methylase	★	★	★	–
	NrdA.1-like protein	★	★	★	–
	ribosyl nicotinamide transporter	★	★	★	–
	putative phosphoesterase	★	★	★	**–**
	NAD-dependent protein deacetylase of the SIR2 family	★	★	★	**–**
	ribonucleotide reductase of class Ia (aerobic) alpha subunit	★	★	★	**–**
	ribonucleotide reductase of class Ia (aerobic) beta subunit	★	**–**	**–**	**–**
	ribonucleotide reductase of class III (anaerobic) large subunit	★	★	★	**–**
	ribonucleotide reductase of class III (anaerobic) activating protein	★	★	★	**–**
	ClpP ATP-dependent protease subunit	★	★	★	**–**
	cell wall hydrolase SleB	★	★	★	**–**
	F-box domain protein	–	●	●	**–**
	putative phosphatase	–	●	●	**–**

The large terminase subunit and putative baseplate assembly protein were only present in the DNA packaging of Group I phages, which displayed endonuclease activity and were involved in DNA translocation and chromosomal end formation during replication. Indeed, the large terminase subunits participated in the packing process through the single-stranded cohesive ends strategy. Thus, highly conserved characteristics in caudoviruses have frequently been used to infer phylogeny (Liu et al., 2018).

Ten DNA manipulation genes, predominantly DNA replication and regulation-related genes, were identified. Both phage groups had DNA replicative helicase/primase and DNA polymerase-related genes; however, Group II phages had six unique DNA replication and regulation genes, including DNA maturase B, ATP-dependent DNA ligase, tRNA nucleotidyltransferase/poly (A) polymerase, DNA exonuclease, DNA endonuclease VII, and DNA-dependent RNA polymerase. These genes are involved in regulating nucleic acid biosynthesis and metabolism. Moreover, Group I phages implemented DNA replication machinery using six other genes, suggesting that the DNA replication machinery of phages was based on diverse strategies and enriched with encoding DNA replication protein. Additionally, phages EspYZU14 and EspYZU15 in Group I (but not EspYZU12) had EndoVII packaging and recombination endonuclease, whose role is to correct deficiencies in genetic recombination and DNA repair.

Genome-wide phage sequences can analyze phage enzymes as potential candidates for active antimicrobial agents. Phage lytic enzymes were identified as potential novel biocontrol agents (Grabowski et al., 2021). These phage enzymes were detected in the whole genomes of two groups of phages (Fig. S2). After DNA replication and assembling new phages, phages usually rely on a lysis system to release phage progeny from the host. A lysis system called the endolysin-holin system is represented in the group II phage lysis system and consists of two lytic glycosylases in group II, namely holin and endolysin. Among them, the lytic glycosylases and endolysins responsible for degrading the bacterial peptidoglycan cell wall had 87.7% and 85.2% homology with *Cronobacter* phage vB_CskP_GAP227 (AFY63158.1) and *Cronobacter* phage Dev-CD-23,823 (CUH74617.1), respectively. Holin, which can subsequently promote the entry of endolysin into the cell wall, leading to rapid cleavage of the cell wall and cell lysis, showed 81.8% homology with *Cronobacter* phage Dev-CD-23,823. In contrast, another lysis system was observed in group I phages. It is widely acknowledged that the lytic enzyme acts alone and is similar to the cortical acid-degrading protein in group I phages. This phage lytic enzyme had 94% homology to *Escherichia* phage 4MG (AGZ17521.1).

Other functional genes were involved in completing the growth cycle of phages (only in Group I). However, there were marginal differences between EspYZU12 and EspYZU14 (EspYZU15) in Group I phages. For instance, ribonucleotide reductase of the class Ia (aerobic) beta subunit was only detected in EspYZU12 but had no F-box domain protein and putative phosphatase, which may result in differences in controlling lytic replication.

The functional genes shown in Table 2 play an essential role in the growth cycle of phages, including adsorption, injection, replication, assembly, and release. Although group phages with different genes may play similar functional roles, their infection strategies and proliferation pathways could be distinct, which might be related to a different host bacteria or sources. In addition, the phages had no virulence genes, indicating the safe profile of the four phages.

#### 3.7.3 Phylogenetic analysis of phages

**Fig. 7:**
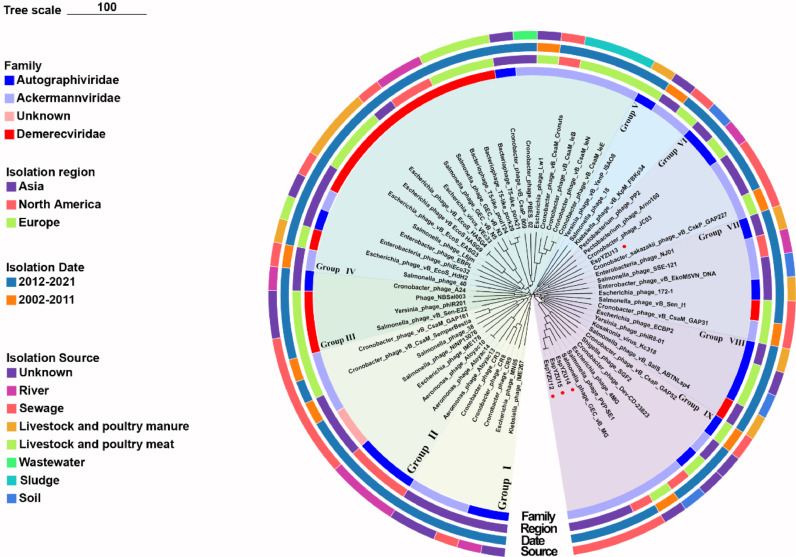
The phylogenetic tree of four phage isolates and 56 *Enterobacter* phages was based on complete genome sequence clusters. The four circles are colored (from inside to outside) by family, isolation region, isolation date, and isolation source. The bootstrap values of branches color the groups.

The Genome-Blast Distance Phylogeny method was utilized to analyze the evolutionary relationship of the four new *C. sakazakii* and 56 *Enterobacter* phages with high homology from the NCBI GenBank database (Bozdeveci et al., 2021). All phages were classified using the bootstrap values of branching across individuals or clusters (Fig. 7). The phylogenetic tree revealed that sixty phages belonged to the *Ackermannviridae* (23), *Autographiviridae* (20), *Demerecviridae* (15), families, and 2 phages were not identified. Notably, all selected phages were distributed across nine groups of the phylogenetic tree rather than in a clustered group (Group I to Group IX).

Similarly, phages from different isolation origins were analyzed in this study. EspYZU12 (containing EspYZU14 and EspYZU15) in group IX had the highest identity (94.6% identity and 83.5% coverage) with *Salmonella* phage GEC_vB_MG (*Salmonella* phage PVP-SE1) from the sewage of Asia-Europe. Besides, EspYZU13 in Group VI had the highest homology with *Cronobacter* phage vB_CskP_GAP227 (97.84% homology and 95% coverage) from the sewage of Asia, which indicated a significant difference between the genomes and the tremendous evolutionary distances among each *Cronobacter* phage.

#### 3.7.4 Comparative genomic analysis

**Fig. 8:**
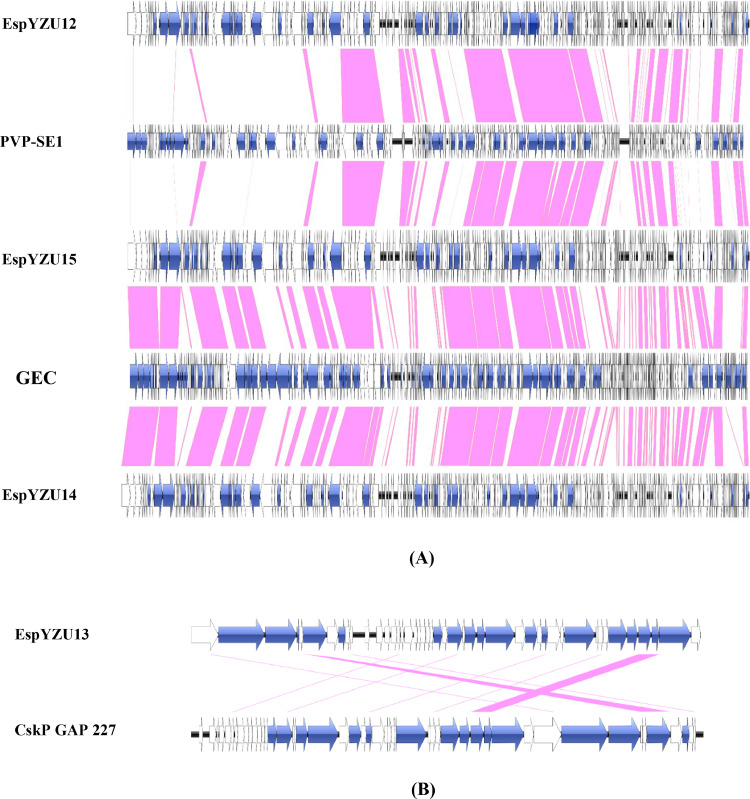
Comparative structural analyses of the phages EspYZU12, EspYZU15, EspYZU14, PVP-SE1, GEC (A), EspYZU13, and CskP GAP 227 (B). Each ORF is represented by arrows indicating the size and transcriptional direction. The hypothetical proteins are shown in white, and the known ORFs are shown in blue. The pink-shaded region denotes the part of the nucleotide sequence that is more than 90% homologous.

Comparative genomic analysis was performed on the five phage genomes (Fig. 8A). A total of 131 genes with above 90% identity were homologous between the genome sequence of phage EspYZU12 (EspYZU14 and EspYZU15) and *Salmonella* phage GEC_vB_MG (*Salmonella* phage PVP-SE1), of which 61 genes have been documented, including eight structure-associated genes and 53 nucleic acid metabolism- and expression-associated genes. In other words, eight genes consisted of six putative tail fiber proteins (ORF 32, ORF 37, and ORF60 of EspYZU15 and ORF 34, ORF 39, and ORF 62 of EspYZU14) and two tail sheath proteins (ORF 52 of EspYZU15 and ORF 54 of EspYZU14). The remaining 53 genes associated with nucleic acid metabolism and expression included 17 significant subunit genes, seven putative membrane proteins, three carbohydrate-binding domain proteins, nine DNA- and RNA-associated proteins, three terminase large subunits, three cell wall hydrolase SleB, three ClpP ATP-dependent protease subunits, and eight transport and packing proteins.

Likewise, the EspYZU13 genome was compared to the *Cronobacter sakazakii* phage vB_CskP_GAP227 (KC107834). As delineated in Fig. 8B, all 11 genes showed high homology (> 90% identity) with the EspYZU13 and *Cronobacter sakazakii* phage vB_CskP_GAP227, which included six hypothetical proteins and five functional proteins, two structure-associated genes (ORF42 and ORF43) and three nucleic acid metabolism- and expression-associated genes, namely DNA maturase B (ORF6), DNA helicase (ORF25), and DNA endonuclease VII (ORF33).

## 4 Conclusion

In this study, the morphological and biological properties of four novel *C. sakazakii* phages isolated from sewage samples demonstrated their biocontrol potential against *C. sakazakii* in the industry. In addition, our comparative genomic analysis results provided novel insights into the genetic function, expression mechanism, and evolution of the four *C. sakazakii* phages. To sum up, the four wild lytic phages revealed in this research have huge prospects for application as biocontrol agents.

## CRediT authorship contribution statement

**Yuan-Song Zhang:** Investigation, Writing – original draft. **Lei Yuan:** Validation, Writing – review & editing. **Fedrick C. Mgomi:** Writing – review & editing. **Cao-Wei Chen:** Conceptualization, Methodology. **Yang Wang:** Writing – review & editing. **Zhen-Quan Yang:** Supervision, Project administration. **Xin-an Jiao:** Funding acquisition.

## Declaration of Competing Interest

The authors declared that they have no conflicts of interest to this work.

## Data Availability

Data will be made available on request.

## Appendix Supplementary materials

**Fig. S1.** Genomes circles of EspYZU12 (A), EspYZU13 (B), EspYZU14 (C) and EspYZU15 (D). From the outside, circles display (1) Open reading frames (ORFs) transcribed clockwise or counterclockwise. (2) G + C% content. Values > 50.86% (average) are represented by outward peaks, and inward peaks represent smaller values. (3) GC skew (G - C/ G + C in a 1-kb window and 0.1-kb incremental shift). Values greater than zero are in magenta, and smaller values are in green. (4) Physical map scaled in Kbp. Image, application 1

Image, application 2
