# Supplementary material for: Characterization and comparative genomic analysis of novel lytic bacteriophages targeting Cronobacter sakazakii
Source: Virus Res. 2023 Apr 1;329:199102. doi: 10.1016/j.virusres.2023.199102 (PMC10194406; doi:10.1016/j.virusres.2023.199102)
Supplement: Supplementary file 1 — Fig. S1. Genomes circles of EspYZU12 (A), EspYZU13 (B), EspYZU14 (C) and EspYZU15 (D). From the outside, circles display (1) Open reading frames (ORFs) transcribed clockwise or counterclockwise. (2) G + C% content. Values > 50.86% (average) are represented by outward peaks, and inward peaks represent smaller values. (3) GC skew (G - C/ G + C in a 1-kb window and 0.1-kb incremental shift). Values greater than zero are in magenta, and smaller values are in green. (4) Physical map scaled in Kbp. [file mmc1.docx]

**Figure S1**


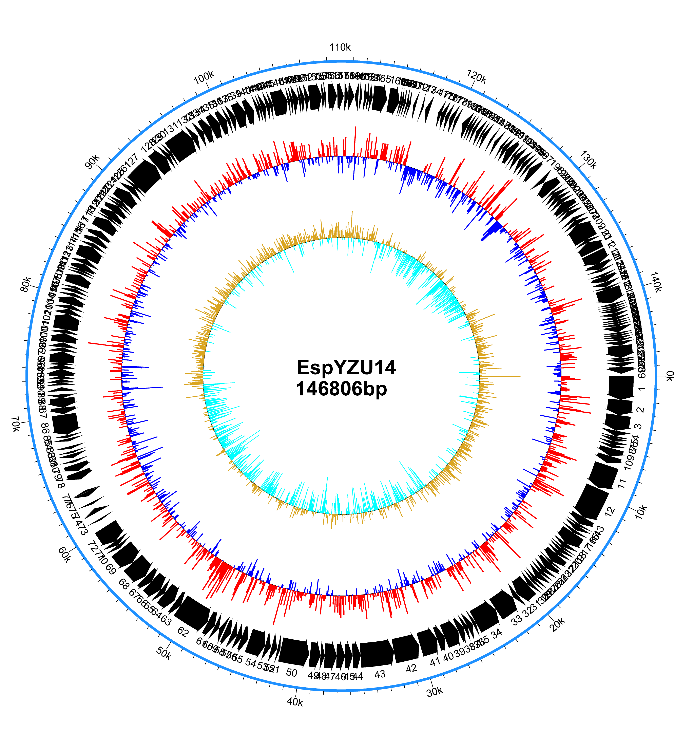

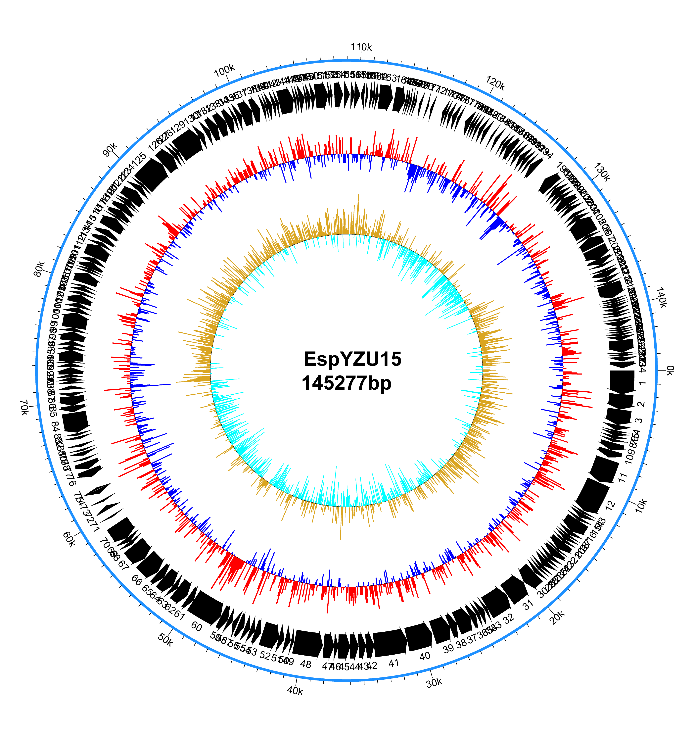


**(B)**

**(C)**

**(D)**


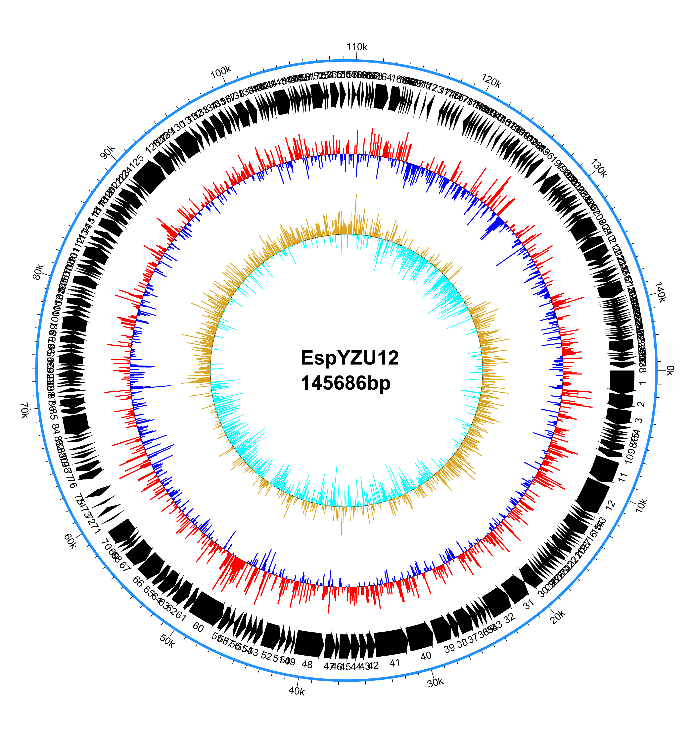


**(A)**


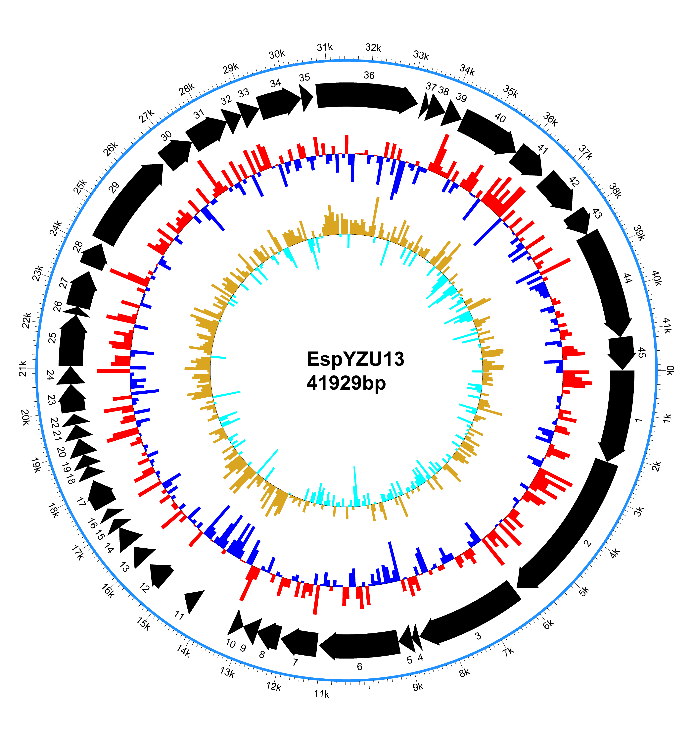


**Fig. S1.** Genomes circles of EspYZU12 (A), EspYZU13 (B), EspYZU14 (C) and EspYZU15 (D). From the outside, circles display (1) Open reading frames (ORFs) transcribed clockwise or counterclockwise. (2) G + C % content. Values > 50.86% (average) are represented by outward peaks, and inward peaks represent smaller values. (3) GC skew (G - C/ G + C in a 1-kb window and 0.1-kb incremental shift). Values greater than zero are in magenta, and smaller values are in green. (4) Physical map scaled in Kbp.

**Figure S2**


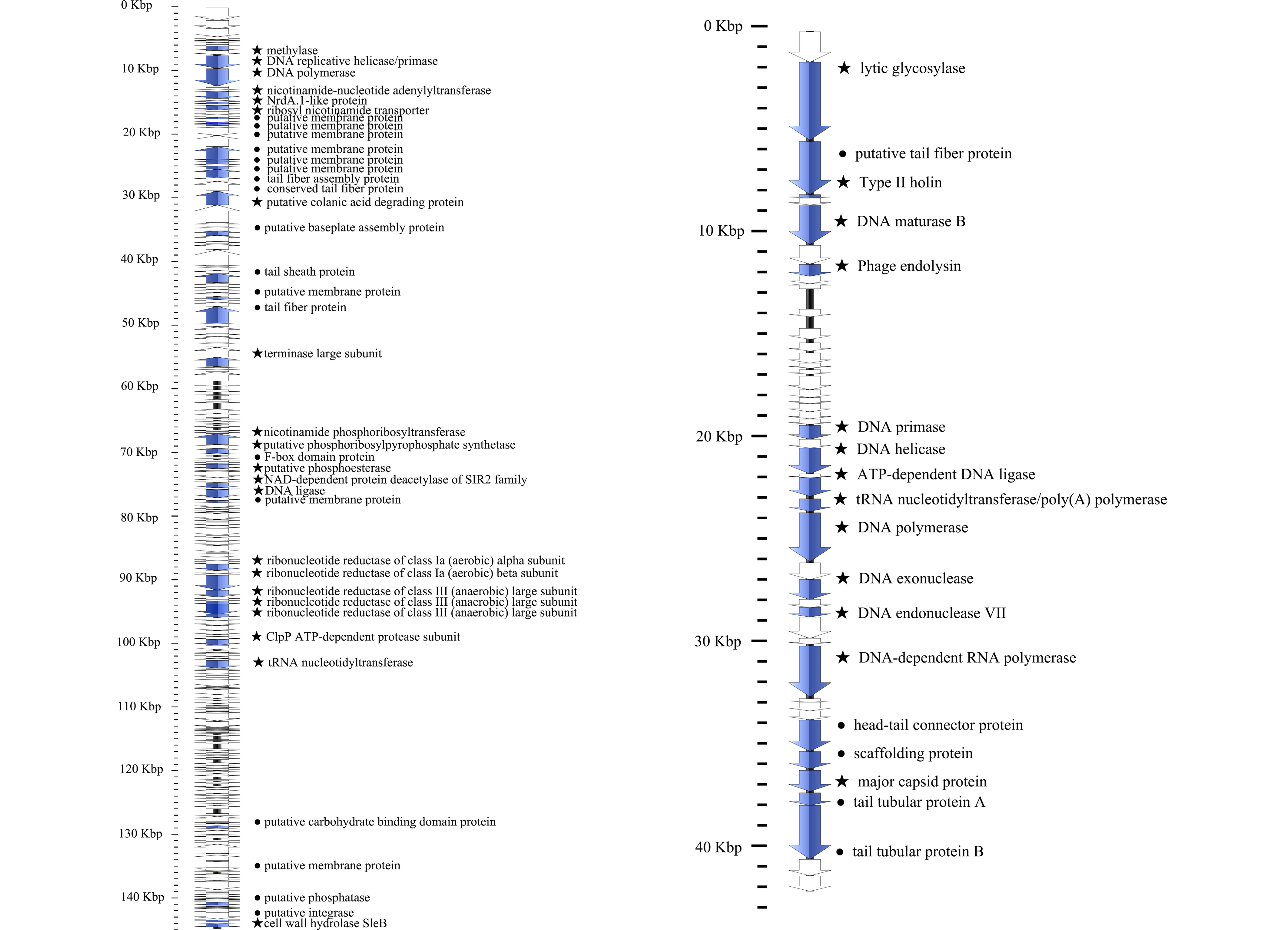


1. **(B)**


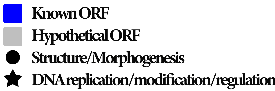

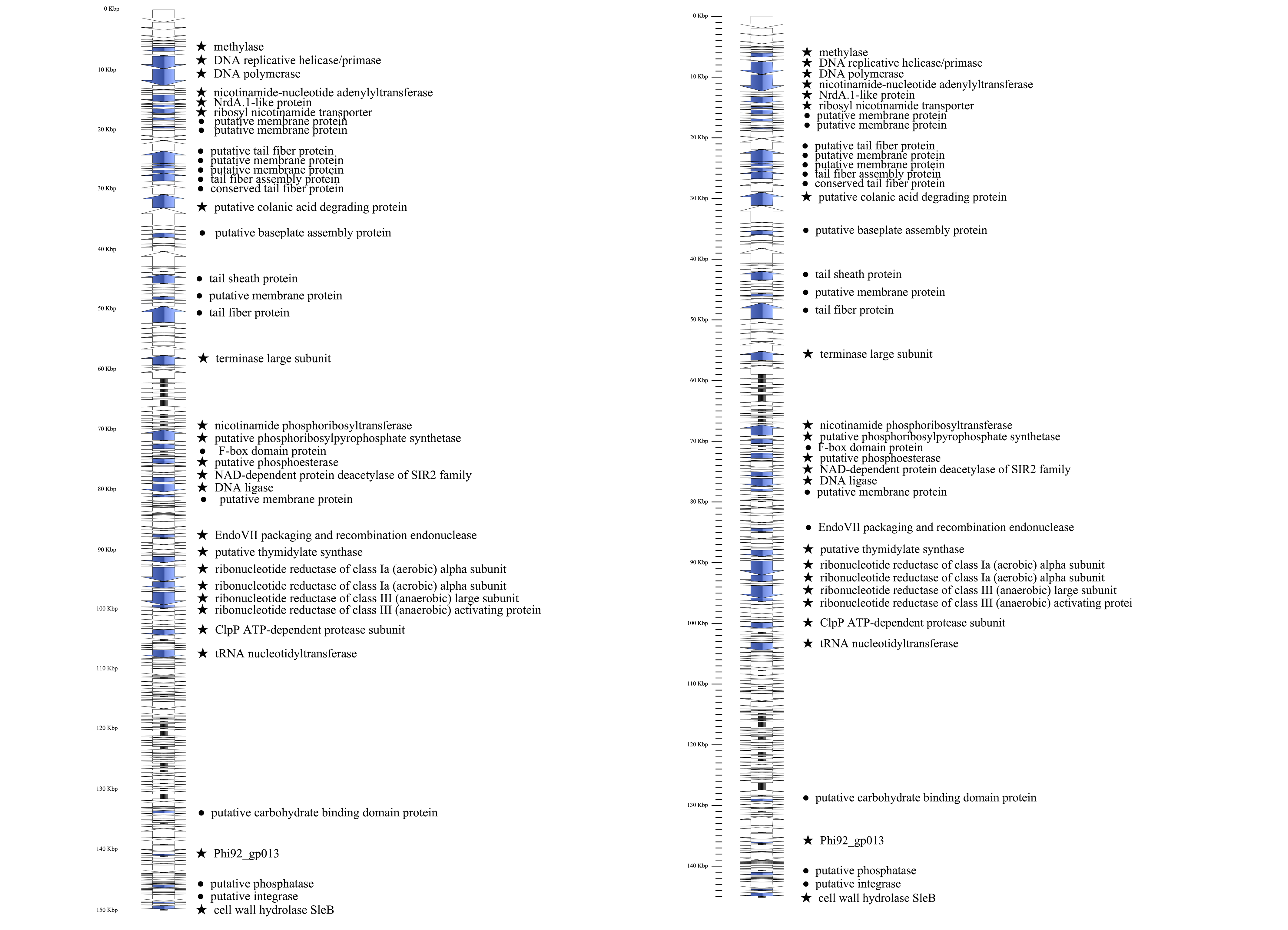


**(D)**

**(C)**

**Fig. S2.** Schematic representation of the linear dsDNA genome of phage EspYZU12 (A), EspYZU13 (B), EspYZU14 (C) and EspYZU15 (D). An arrow with predicted functions represents the positions, orientation, and function of predicted ORFs.
