# Supplementary material for: Characterization and comparative genomic analysis of novel lytic bacteriophages targeting Cronobacter sakazakii
Source: Virus Res. 2023 Apr 1;329:199102. doi: 10.1016/j.virusres.2023.199102 (PMC10194406; doi:10.1016/j.virusres.2023.199102)
Supplement: Supplementary file 2 [file mmc2.docx]

**Table S1** All strains used in this study.

| **No.** | **Species** | **Strain No.** | **Stored** | **Source** |
| --- | --- | --- | --- | --- |
| 1 | *C. sakazakii* | CICC 21560 | China Center of Industrial Culture Collection (CICC) | Unknown |
| 2 |  | CICC 21545 |  | Milk Powder |
| 3 |  | CICC 21569 |  | Chocolate-chip Biscuit |
| 4 |  | CICC 21673 |  | Unknown |
| 5 |  | CICC 22919 |  | Unknown |
| 6 |  | BQW100020180009 | Yang Zhou Public Health Center | Milk Powder |
| 7 |  | BQW100020180010 |  |  |
| 8 |  | BQW100020180011 |  |  |
| 9 |  | BQW100020180012 |  | Clinical Sample |
| 10 |  | BQW100020180013 |  |  |
| 11 | *E. coli* | CICC 10664 | China Center of Industrial Culture Collection (CICC) | Standard strain |
| 12 | *S.* Enteritidis | CICC 21513 |  | Frozen Mizuhopecten yessoensis |
| 13 | *S. aureus* | CICC 21600 |  | Unknown |
| 14 | *B. cereus* | CICC 21261 |  | Unknown |

**Table S2** The lysis spectrum of *Cronobacter* phages.

| No. | Species | Strain No. | Lysis by phage * | | | |
| --- | --- | --- | --- | --- | --- | --- |
|  |  |  | EspYZU12 | EspYZU13 | EspYZU14 | EspYZU15 |
| 1 | *C. sakazakii* | CICC 21560 | - | + | + | - |
| 2 |  | CICC 21545 | + | - | - | - |
| 3 |  | CICC 21569 | - | Host | + | + |
| 4 |  | CICC 21673 | - | + | + | + |
| 5 |  | CICC 22919 | Host | + | Host | Host |
| 6 |  | BQW100020180009 | + | + | - | + |
| 7 |  | BQW100020180010 | - | + | - | - |
| 8 |  | BQW100020180011 | + | + | - | - |
| 9 |  | BQW100020180012 | + | + | + | + |
| 10 |  | BQW100020180013 | + | + | - | + |
| 11 | *E. coli* | CICC 10664 | - | - | - | - |
| 12 | *S.* Enteritidis | CICC 21513 | - | - | - | - |
| 13 | *S. aureus* | CICC 21600 | - | - | - | - |
| 14 | *B. cereus* | CICC 21261 | - | - | - | - |
| Number of strains | | | 6/14 | 9/14 | 5/14 | 6/14 |
| Lytic activity (%) | | | 42.86 | 64.29 | 35.71 | 42.86 |

* +: plaque formation; -: no plaque formation.

**Table S3** Physiological properties of the four phages in this study.

| Phages | Stability test | | burst size  (PFU/cell) | Lytic activity  (Number and %) | Optimal MOI |
| --- | --- | --- | --- | --- | --- |
|  | pH (lowest) | Thermal (℃ and mins) |  |  |  |
| EspYZU12 | 5 | 80 (40) | 250 | 6 (42.86) | 0.001 |
| EspYZU13 | 4 | 80 (40) | 10 | 9 (64.29) | 0.1 |
| EspYZU14 | 5 | 80 (10) | 31 | 5 (35.71) | 0.1 |
| EspYZU15 | 5 | 80 (20) | 96 | 6 (42.86) | 0.001 |

**Table S4** Statistical representation of predicted results of coding genes.

| Sample ID | Genome size (bp) | GC Content (%) | Gene number | Gene total length (bp) | Gene average length (bp) | Gene length / Genome (%) | Accession  Number |
| --- | --- | --- | --- | --- | --- | --- | --- |
| EspYZU12 | 145,686 | 46.72 | 238 | 131,241 | 551 | 90.1 | OP819284 |
| EspYZU13 | 41,929 | 54.71 | 45 | 39,069 | 868 | 93.2 | OP819285 |
| EspYZU14 | 146,806 | 46.71 | 239 | 132,615 | 554 | 90.3 | OP850600 |
| EspYZU15 | 145,277 | 46.74 | 234 | 130,419 | 557 | 89.8 | OP866730 |
